# Gga-miR-3525 Targets *PDLIM3* through the MAPK Signaling Pathway to Regulate the Proliferation and Differentiation of Skeletal Muscle Satellite Cells

**DOI:** 10.3390/ijms21155573

**Published:** 2020-08-04

**Authors:** Huadong Yin, Jing Zhao, Haorong He, Yuqi Chen, Yan Wang, Diyan Li, Qing Zhu

**Affiliations:** Farm Animal Genetic Resources Exploration and Innovation Key Laboratory of Sichuan Province, Sichuan Agricultural University, Chengdu 611130, China; yinhuadong@sicau.edu.cn (H.Y.); zhaojing@stu.sicau.edu.cn (J.Z.); hehaorong@stu.sicau.edu.cn (H.H.); chenyuqi@stu.sicau.edu.cn (Y.C.); wangyan519@sicau.edu.cn (Y.W.); diyanli@sicau.edu.cn (D.L.)

**Keywords:** skeletal muscle satellite cells (SMSCs), gga-miR-3525, PDLIM3, MAPK pathway, proliferation, differentiation

## Abstract

MicroRNAs (miRNAs) are evolutionarily conserved, small noncoding RNAs that post-transcriptionally regulate expression of their target genes. Emerging evidence demonstrates that miRNAs are important regulators in the development of skeletal muscle satellite cells (SMSCs). Our previous research showed that gga-miR-3525 is differentially expressed in breast muscle of broilers (high growth rate) and layers (low growth rate). In this study, we report a new role for gga-miR-3525 as a myogenic miRNA that regulates skeletal muscle development in chickens. Exogenous increases in the expression of gga-miR-3525 significantly inhibited proliferation and differentiation of SMSCs, whereas the opposite effects were observed in gga-miR-3525 knockdown SMSCs. We confirmed that *PDLIM3* (PDZ and LIM domain 3) is a target gene of gga-miR-3525 that can promote proliferation and differentiation of SMSCs. We found that *PDLIM3* overexpression elevated the abundance of phosphorylated (p-)p38 protein but that the gga-miR-3525 mimic and p38-MAPK inhibitor (SB203580) weakened the activation of p-p38. Furthermore, treatment with SB203580 reduced the promoting effect of *PDLIM3* on SMSC proliferation and differentiation. Overall, our results indicate that gga-miR-3525 regulates the proliferation and differentiation of SMSCs by targeting *PDLIM3* via the p38/MAPK signaling pathway in chickens.

## 1. Introduction

Skeletal muscle is one of the most abundant tissues in humans and animals (40–60% of the body weight of adult animals). Healthy skeletal muscle has a positive effect on the normal progress of physical activities such as exercise and breathing. Any deviation from normal function can lead to disease, including cancer and diabetes [1]. Skeletal muscle is the main component of edible meat and the primary source of animal protein in the world. The quantity and quality of skeletal muscle affects the quality of meat products and directly determines the economic value of animals [2]. The number of animal muscle fibers is generally fixed in the embryonic stage. Skeletal muscle development after birth is mainly based on the proliferation, differentiation, and fusion of activated skeletal muscle satellite cells (SMSCs) to form new muscle fibers and promote the proliferation of muscle fibers [3].

The development of SMSCs is a complex process regulated by many signaling pathways, genes, and noncoding RNAs [4]. In recent years, following extensive research into microRNAs (miRNAs; a type of small noncoding RNA), an increasing number of miRNAs have been identified as participating in muscle development, enriching our understanding of intricate skeletal muscle development networks [5]. MiRNAs contain approximately 22 nucleotides and usually bind to the seed sequence of target genes and negatively regulate their expression after transcription [6,7]. MiRNAs bind to target mRNAs through seed sequences, and reduce the expression of target genes by inhibiting mRNA translation or promoting mRNA decay. It is well known that miRNAs play different roles in basic biological processes, including cell proliferation, differentiation, disease occurrence, and formation of tissues and organs [8,9].

By analyzing our previous miRNA-Seq and RNA-Seq data (NCBI accession number PRJNA516545), we found that gga-miR-3525 was differentially expressed in chickens with different muscle growth rates (*p* < 0.05, broilers and layers). Sequencing and real-time quantitative polymerase chain reaction (RT-qPCR) data showed that the expression of gga-miR-3525 in broilers (high growth rate) was lower than that in layers (low growth rate), suggesting that gga-miR-3525 may have a negative effect on skeletal muscle development [10]. gga-miR-3525 (miRBase accession number: MIMAT0016382) is specifically expressed in chickens [11]; it was first identified in the lungs and trachea of chickens infected with avian influenza virus and found to target the interleukin-6 signal converter (IL6ST) [12]. Chen et al. found that gga-miR-3525 was involved in probiotics-induced reduction of cecal inflammation by *Salmonella* Typhimurium in neonatal broiler chickens [13]. Additionally, expression of gga-miR-3525 is altered after heat stress in layers, suggesting that gga-miR-3525 may participate in regulation of heat stress-induced damage in the chicken [14]. However, until now, the role of gga-miR-3525 in the chicken skeletal muscle has not been reported.

The aim of the present study was to evaluate the regulatory mechanism of gga-miR-3525 in the proliferation and differentiation of chicken SMSCs. We found that gga-miR-3525 participated in skeletal muscle myogenesis by inhibiting *PDLIM3* (PDZ and LIM domain 3) through the p38/MAPK pathway in chickens.

## 2. Results

### 2.1. gga-miR-3525 Reduced the Proliferation of Chicken SMSCs

**Figure 1:**
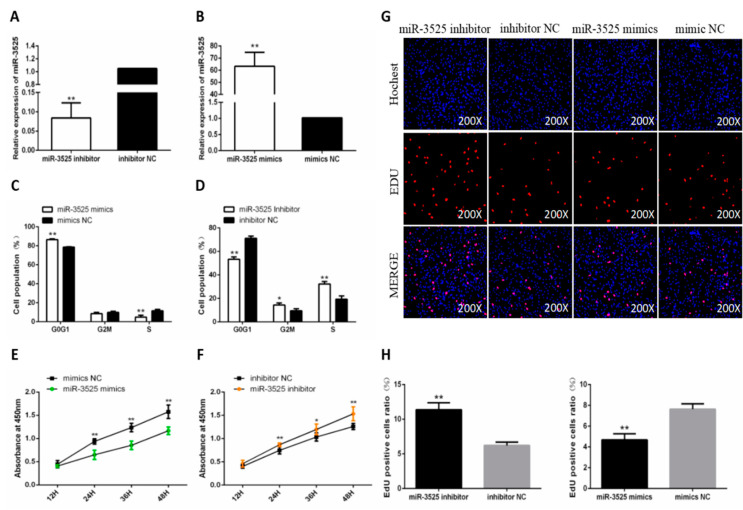
Effect of gga-miR-3525 on the proliferation of skeletal muscle satellite cells (SMSCs). Expression of gga-miR-3525 in SMSCs following transfection with its inhibitor (**A**) or mimic (**B**). Cell cycle analysis of SMSCs after overexpression (**C**) or inhibition (**D**) of gga-miR-3525 for 48 h. Cell growth curves of SMSCs measured by the CCK-8 kit after overexpression (**E**) or inhibition (**F**) of gga-miR-3525. (**G**) Results of EdU assay for SMSCs after overexpression or inhibition of gga-miR-3525 for 48 h, where EdU (red) fluorescence is used as an indicator of proliferation and nuclei are indicated by Hochest (blue) fluorescence. (**H**) EdU-positive cell ratio of SMSCs after overexpression or inhibition of gga-miR-3525 for 48 h. Data are expressed as mean ± SEM (*n* = 6). * *p* < 0.05; ** *p* < 0.01 vs. NC (negative control).

In order to determine the role of gga-miR-3525 in the proliferation and differentiation of SMSC, we first constructed gga-miR-3525 inhibitor and gga-miR-3525 mimic, and verified their efficiency (Figure 1A,B). To determine the effect of gga-miR-3525 on the proliferation of chicken SMSCs, we first examined the cell cycle by flow cytometry. The results showed that the number of G_0_/G_1_ cells increased after addition of gga-miR-3525 mimics, whereas the number of cells in the S phase decreased (Figure 1C). Inhibition of gga-miR-3525 expression reduced the number of G_0_/G_1_ cells (Figure 1D). Subsequent analysis using the CCK-8 kit to detect cell activity showed the same trend: increased expression of gga-miR-3525 decreased cell proliferation activity. Conversely, inhibition of gga-miR-3525 had a positive effect on cell proliferation activity (Figure 1E,F). Next, we further examined the proliferation of SMSCs using the EdU assay. The result showed that knockdown of gga-miR-3525 significantly increased the number of EdU-positive cells, whereas overexpression of gga-miR-3525 reduced the number of cells (*p* < 0.05, Figure 1G,H). Together, these results suggested that gga-miR-3525 inhibits proliferation of chicken SMSCs.

### 2.2. gga-miR-3525 Reduced the Differentiation of Chicken SMSCs

**Figure 2:**
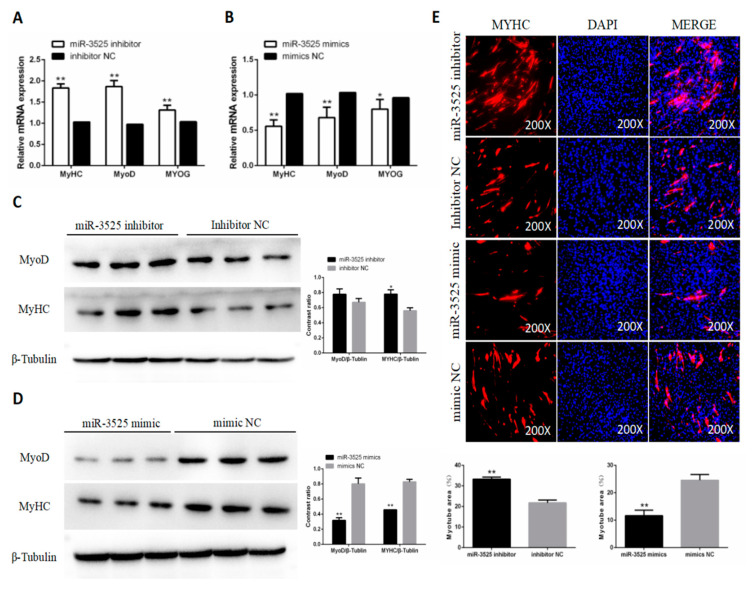
Effect of gga-miR-3525 on differentiation of skeletal muscle satellite cells (SMSCs). mRNA expression of MyHC, MyoD, and MyoG in gga-miR-3525 knockdown (**A**) and overexpressed (**B**) SMSCs. Protein levels of MyoD and MyHC in SMSCs after inhibition (**C**) and overexpression (**D**) of gga-miR-3525. (**E**) Representative images of immunofluorescent staining of differentiated SMSCs. Myosin (red), a molecular marker of myogenesis; DAPI (blue), cell nuclei; Merge: the fusion of SMSCs into primary myotubes. Myotube area (%) in SMSCs after inhibition and overexpression of gga-miR-3525. Data are expressed as mean ± SEM (*n* = 6). * *p* < 0.05; ** *p* < 0.01 vs. NC (negative control).

To explore the role of gga-miR-3525 on SMSC differentiation, we detected expression of the differentiation marker genes myoblast determination protein 1 (MyoD1), myosin heavy chain (MyHC), and myogenin (MyoG). Inhibition of gga-miR-3525 up-regulated the mRNA expression of these three markers and the protein abundance of MyoG and MyHC (*p* < 0.05, Figure 2A,C), and after overexpression of gga-miR-3525 in SMSC, mRNA expression and protein abundance is reduced. (*p* < 0.05, Figure 2B,D). Overexpression of gga-miR-3525 inhibited myotube formation, whereas the addition of gga-miR-3525 inhibitor promoted myotube formation (Figure 2E). Together, these results suggested that gga-miR-3525 inhibits the differentiation of chicken SMSCs.

### 2.3. gga-miR-3525 Can Target PDLIM3 and Inhibit its Expression

**Figure 3:**
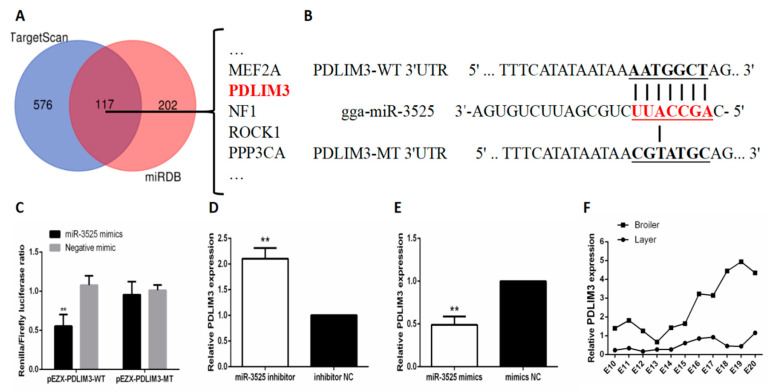
*PDLIM3* as a target gene of gga-miR-3525. (**A**) Prediction of target genes of gga-miR-3525 using TargetScan and miRDB. (**B**) Dual-luciferase reporter gene (pEZX-FR02) with wild type (pEZX-PDLIM3-WT) or mutant (pEZX-PDLIM3-MT). Bold and underlined in red is the seed sequence of gga-miR-3525, and the corresponding black is the binding site of PDLIM3. (**C**) Luciferase assays were performed in DF-1 cells by co-transfection of wild-type or mutant PDLIM3 3′-UTR with a gga-miR-3525 mimic or mimic-NC in skeletal muscle satellite cells (SMSCs). mRNA expression of PDLIM3 in SMSCs after inhibition (**D**) and overexpression (**E**) of gga-miR-3525. (**F**) PDLIM3 expression in pectoral muscles of layers and broilers from the tenth to the twentieth day of embryonic period. Data are expressed as mean ± SEM (*n* = 6). ** *p* < 0.01 vs. NC (negative control).

To further understand the mechanism by which gga-miR-3525 regulates the proliferation and differentiation of SMSCs, we used online software tools (TargetScan and miRDB) to predict the target genes of gga-miR-3525 (Figure 3A). Among the predicted genes, we selected *PDLIM3* for subsequent verification, because it contains a LIM domain, indicating that it may play a role in myogenesis. To verify the targeting relationship between gga-miR-3525 and *PDLIM3* in chicken SMSCs, we conducted a dual luciferase reporter experiment. The dual-luciferase reporter genes of wild-type (pEZX-PDLIM3-WT) and mutant (pEZX-PDLIM3-MT) were constructed from the *PDLIM3* 3′-untranslated region (UTR) sequence (Figure 3B). The dual luciferase reporter gene and gga-miR-3525 mimic were co-transfected into DF1 cells, and activity of firefly luciferase was detected after transfection of wild-type plasmid. However, no changes in firefly luciferase were detected in cells co-transfected with the mutant plasmid (Figure 3C). In addition, after adding gga-miR-3525 inhibitor, the mRNA expression of PDLIM3 increased. On the contrary, after adding exogenous gga-miR-3525 to SMSCs, the mRNA expression of PDLIM3 decreased. (*p* < 0.05, Figure 3D,E). From the comparison of the expression of PDLIM3 in the muscles of layer and broiler chickens from the tenth to the twentieth day of embryonic period, the expression of PDLIM3 in the breast muscle of broilers is higher, which is exactly the opposite of our previous measurement of gga-miR-3525 (Figure 3F). Together, these results suggested that *PDLIM3* is the target gene of gga-miR-3525.

### 2.4. PDLIM3 Promotes the Proliferation of Chicken SMSCs

**Figure 4:**
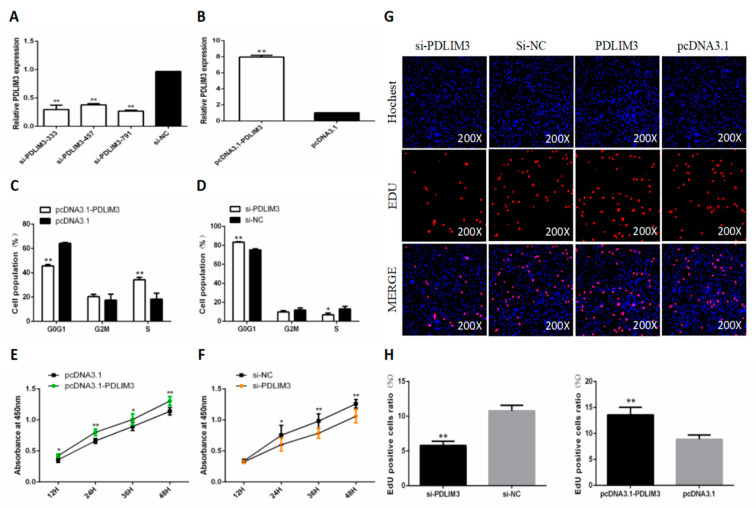
Effect of PDLIM3 on the proliferation of skeletal muscle satellite cells (SMSCs). Expression of miR-PDLIM3 in SMSCs following transfection with its inhibitor (**A**) or mimic (**B**). Cell cycle analysis of SMSCs after overexpression (**C**) or inhibition (**D**) of PDLIM3 for 48 h. Cell growth curves of SMSCs measured by the CCK-8 kit after overexpression (**E**) or inhibition (**F**) of PDLIM3. (**G**) Results of EdU assay for SMSCs after overexpression or inhibition of PDLIM3 for 48 h, where EdU (red) fluorescence is used as an indicator of proliferation and nuclei are indicated by Hochest (blue) fluorescence. (**H**) EdU-positive cell ratio of SMSCs after overexpression or inhibition of PDLIM3 for 48 h. Data are expressed as mean ± SEM (*n* = 6). * *p* < 0.05; ** *p* < 0.01 vs. NC (negative control).

To determine the role of PDLIM3 in SMSC proliferation and differentiation, we constructed and validated PDLIM3 overexpression vectors and siRNA (Figure 4A,B). As we did for gga-miR-3525, we explored the role of PDLIM3 in SMSC proliferation. Flow cytometry revealed that knocking down expression of PDLIM3 in SMSCs increased the proportion of cells in G_0_/G_1_ phase and decreased the proportion of cells in S phase. Overexpression of PDLIM3 produced the opposite results (Figure 4C,D). The CCK-8 results confirmed the flow cytometry results: in SMSC transfected with PDLIM3 overexpression vector, cell proliferation was significantly increased, and in cells expressing PDLIM3 siRNA, cell proliferation was significantly reduced (Figure 4E,F). The trend of the number of EdU-positive cells after the addition of PDLIM3 overexpression plasmid and siRNA was consistent with this conclusion (Figure 4G,H). Together, these results suggested that PDLIM3 promotes proliferation of chicken SMSCs.

### 2.5. PDLIM3 Promotes the Differentiation of Chicken SMSCs

**Figure 5:**
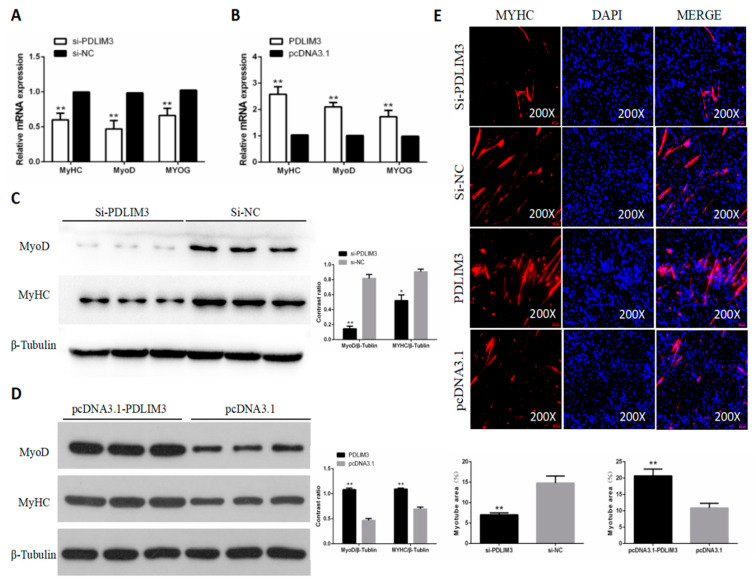
Effect of PDLIM3 on differentiation of skeletal muscle satellite cells (SMSCs). mRNA expression of MyHC, MyoD, and MyoG in PDLIM3 knockdown (**A**) and overexpression (**B**) in SMSCs. Protein levels of MyoD and MyHC in SMSCs after inhibition (**C**) and overexpression (**D**) of PDLIM3. (**E**) Representative images of immunofluorescent staining of differentiated SMSCs. Red: myosin, a molecular marker of myogenesis; blue: DAPI (cell nuclei); Merge: fusion of SMSCs into primary myotubes. Myotube area (%) in SMSCs after inhibition and overexpression of PDLIM3. Data are expressed as mean ± SEM (*n* = 6). * *p* < 0.05; ** *p* < 0.01 vs. NC (negative control).

To study the role of PDLIM3 in differentiation of chicken SMSCs, we assessed the changes in mRNA and protein expression of differentiation marker genes and the formation of myotubes when overexpressing or reducing expression of PDLIM3. We found that after knocking down the expression of PDLIM3 in SMSCs, the mRNA expression and protein abundance of differentiation marker genes were also suppressed (Figure 5A,C). On the contrary, after overexpression of PDLIM3 expression, the mRNA expression and protein abundance of marker genes increase (Figure 5B,D). Immunofluorescence assays confirmed these results. The changes in myotube area observed after PDLIM3 interference and overexpression were consistent with this trend (Figure 5E). Together, these results showed that PDLIM3 positively affected the differentiation of chicken SMSCs.

### 2.6. Transcriptomic Changes Induced by PDLIM3

**Figure 6:**
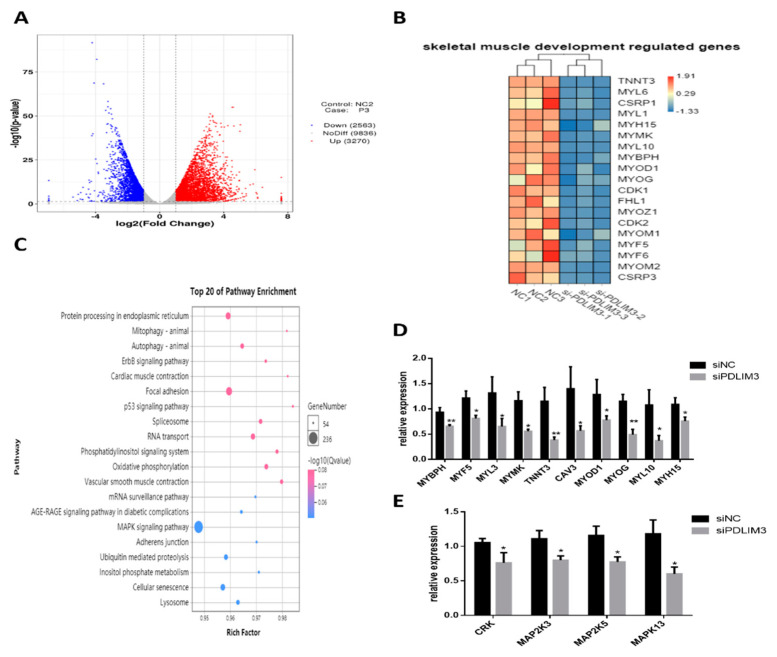
Transcriptomic changes in skeletal muscle satellite cells (SMSCs) after PDLIM3 inhibition. (**A**) Volcano maps showing 3270 upregulated and 2563 downregulated genes based on RNA-seq analysis of samples transfected with PDLIM3 siRNA. (**B**) Heat map clustering of differentially expressed genes related to muscle development in samples transfected with PDLIM3 siRNA. (**C**) Top 20 pathways of the differentially expressed genes according to KEGG enrichment analysis. mRNA expression of genes involved in muscle development (**D**) and the MAPK pathway (**E**) in samples transfected with PDLIM3 siRNA or in negative controls (siNC). Data are expressed as mean ± SEM (*n* = 6). * *p* < 0.05; ** *p* < 0.01 vs. NC (negative control).

We performed RNA-Seq analysis on samples transfected with PDLIM3 siRNA for 48 h (NCBI accession number: GSE151450). The results showed that there were 5833 differentially expressed genes between treatment and control. Of these, 3270 genes were upregulated and 2563 were downregulated (Figure 6A). In the interference group, expression of some genes that promote proliferation and differentiation, such as *CDK1*, *CDK2*, *MYH15*, *MyoD1*, *MyoG*, *MYF5*, and *MYF6*, was significantly reduced (*p* < 0.05, Figure 6B). The KEGG pathway results showed that these differentially expressed genes were mainly enriched in 23 pathways (*p* < 0.05). Most of the differentially expressed genes were found to be related to important biological processes, including the cGMP-PKG signaling pathway, oxytocin signaling pathway, MAPK signaling pathway, tight junction, mammalian (or mechanistic) target of rapamycin (mTOR), and other signaling pathways (Figure 6C). To verify the transcriptome sequencing results, we verified the mRNA expression of genes related to muscle growth and development, as well as key genes of the MAPK signaling pathway and actin cytoskeleton p38. Changes in the expression of these genes after interfering with PDLIM3 were consistent with the results obtained by transcriptome sequencing (Figure 6D,E).

### 2.7. gga-miR-3525 Regulates Proliferation and Differentiation through the p38/MAPK Pathway

**Figure 7:**
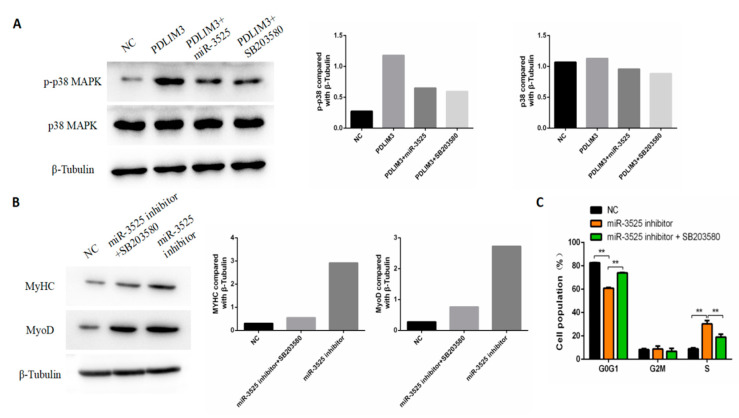
gga-miR-3525 regulates proliferation and differentiation through the p38/MAPK pathway in skeletal muscle satellite cells (SMSCs). (**A**) Protein level of p38 and phosphorylated (p)-p38 in SMSCs transfected with PDLIM3, PDLIM3 + gga-miR-3525, and PDLIM3 + SB203580 (p38-MAPK inhibitor), respectively. Effect of gga-miR-3525 knockdown on proliferation (**B**) and differentiation (**C**) of SMSCs in the absence or presence of SB203580. Data are expressed as mean ± SEM (*n* = 6). ** *p* < 0.01 vs. NC (negative control).

Next, we explored whether gga-miR-3525 regulates the proliferation and differentiation of SMSCs through the p38-MAPK pathway. We measured the abundance of phosphorylated p38 protein (p-p38) and total p38 protein (t-p38). As shown in Figure 7A, the expression of p-p38 was activated after adding the PDLIM3 plasmid, but the level of p38 did not change. Interestingly, co-transfection of gga-miR-3525 mimics or p38-MAPK inhibitor (SB203580) into SMSCs alleviated the activation of p-p38. After interfering with gga-miR-3525, the ratio of cells in G_0_/G_1_ relative to cells in S phase was reduced and the protein abundance of markers of differentiation was increased. However, after co-transfection of SB203580, these trends showed a certain degree of relaxation (Figure 7B,C). Taken together, these results indicate that gga-miR-3525 regulates the p38-MAPK pathway by inhibiting PDLIM3.

## 3. Discussion

The chicken is known to be an ideal model for studying the formation of vertebrate skeletal muscle; its embryonic development in vitro has advantages of ease of operation and low cost for chicken embryo research and development, and studies in chicken have led to some major discoveries in developmental biology [15]. Skeletal muscle is the most abundant tissue in vertebrates. Together with the bones, it forms the movement system for humans and animals. With the rapid discovery of essential genes, chickens are useful models for testing functions. Revealing the molecular basis of development provides a direct link to clinical genetics. Therefore, basic research on chickens is of great significance for understanding human health and disease [16].

In this study, CCK-8 and EdU analyses showed that downregulation of gga-miR-3525 promoted the proliferation of SMSCs, whereas upregulation of gga-miR-3525 had the opposite effect. This indicates that gga-miR-3525 can inhibit the proliferation of chicken SMSCs. Additionally, by analyzing the changes in expression of myogenic differentiation marker genes and myotube formation, we found that gga-miR-3525 inhibits the differentiation of SMSCs. In the miRBase database, miR-3525 is unique among chickens. Previous studies on gga-miR-3525 focused on lung, trachea [12], and cecum [13]. To the best of our knowledge, this is the first study to examine its role in skeletal muscle development.

We use DAVID Bioinformatics Resources 6.8 (https://david.ncifcrf.gov/) to annotate the potential target genes of gga-miR-3525 [17]. Screening the potential genes revealed genes related to muscle development, including *MEF2A* (myocyte enhancing factor 2A), *PDLIM3*, *NF1* (neurofibrin 1), *ROCK1* (Rho-related coiled-coiled protein), and *PPP3CA* (protein phosphatase 3, catalytic subunit, alpha isozyme). Interestingly, we found that PDLIM3 contains a LIM domain. Genes containing LIM domains usually have a role in the process of myogenesis [18]. PDLIM3, also known as ALP (actin-associated LIM protein), is a member of the PDZ-LIM domain family [19]. It is mainly composed of a N-terminal PDZ domain and a C-terminal LIM domain, and it is expressed in skeletal, cardiac, and smooth muscle. It co-localizes with α-actinin on the Z line of mature muscle fibers [20]. Studies have shown that all PDZ and LIM domain-encoded proteins can bind to or affect the actin cytoskeleton, and several PDZ-LIM proteins are mainly expressed in skeletal muscle [21]. Abnormal selective splicing of this gene in human skeletal muscle plays a part in myotonic dystrophy and, after abnormal splicing, the gene has been found to regulate skeletal muscle development [22]. Pomiès et al. reported that the expression of PDLIM3 subtype is accompanied by a dramatic increase in C2C12 differentiation and concluded that PDLIM3 plays a major role in maintaining the differentiation of C2C12 myoblasts [23]. Another study showed that PDLIM3 can enhance the cross-linking ability of β-actinin in actin filaments and play a role in regulating the activity of serum response factor (SRF) in the cytoskeleton [24]. A new PDLIM3 splice variant was identified in porcine skeletal muscle that is expressed only in heart and skeletal muscle, showing the highest expression in adult porcine skeletal muscle [25]. Piórkowska et al. concluded that *PDLIM3* is a candidate gene for pork quality through quantitative trait loci [26]. These results indicate that PDLIM3 plays important roles in maintaining muscle cell stability, cell differentiation, cell signal transduction, cell proliferation, and cytoskeletal structure integration and that it has a positive role in muscle development. In the research we have reported, RNA-Seq and RT-qPCR show that gga-miR-3525 is likely to play an inhibitory role in myogenesis [10]. Therefore, because of the mechanism of action of miRNA, we chose *PDLIM3* as a target gene of gga-miR-3525 for subsequent verification. The results of the dual luciferase reporter assay conducted herein confirmed that PDLIM3 is a target gene of gga-miR-3525. A subsequent series of experiments confirmed that PDLIM3 had a positive effect on the proliferation and differentiation of SMSCs.

Members of the mitogen-activated protein kinase (MAPK) signaling pathway family include the c-Jun NH2-terminal kinase (JNK) family (also known as stress-activated protein kinase), extracellular regulatory kinase (ERK) family, and p38 family. For the most part, the JNK/MAPK pathway is significantly downregulated in the late stage of myogenesis and negatively regulates skeletal muscle differentiation, whereas the p38/MAPK pathway is upregulated [27]. The p38 family includes p38α, p38β, p38γ, and p38δ signaling pathways. P38α is significantly expressed in most cell types, whereas p38β, p38γ, and p38δ are mainly expressed in the brain, skeletal muscle, and endocrine glands, respectively [28]. The p38α/β MAPK signaling pathway participates in the asymmetric division of SMSCs and the differentiation of SMSCs in vivo; it is necessary for the activation of satellite cells and can regulate the resting state of satellite cells [29]. Further research has shown that the p38 MAPK signaling pathway is the basis for the self-renewal of aging skeletal muscle stem cells, a discovery that suggests potential for its therapeutic application in progressive muscle wasting [30]. The MAPK signaling pathway is known to play an important role in skeletal muscle development, and evidence indicates that the p38 signaling pathway plays a key role in the activation of MEF2 transcription factors during mouse somatic development. The early expression of Myf5 in *Xenopus laevis* and the expression of several muscle structural genes have also been shown to require the p38 signaling pathway [31]. Wang et al. showed that miR-487b-3p targets IRS1, an important regulator in the MAPK pathway, to affect mouse myogenesis [32]. Through functional network analysis of differentially expressed miRNA and mRNA between sex-linked dwarf (SLD) and normal chickens, Luo et al. found that the MAPK pathway may play a critical role in the loss of muscle mass caused by growth hormone receptor (GHR) deficiency [33]. In this study, by analyzing RNA-Seq results of si-PDLIM3 cells and normal cells, we found that expression levels of multiple genes in the MAPK pathway were altered; these results were confirmed by the RT-qPCR results. KEGG pathway analysis showed significant enrichment of genes in the MAPK pathway. Therefore, we further explored the role of the MAPK pathway in gga-miR-3525 and PDLIM3 in affecting the proliferation and differentiation of SMSCs. p38 MAPK activation is an important mechanism of myogenesis; we observed that p38 MAPK was activated by exogenous PDLIM3 plasmid during myogenesis, whereas gga-miR-3525 mimics and the p38 MAPK inhibitor SB203580 stopped the process. Additionally, specific inhibitors effectively prevented the proliferation and differentiation of SMSC cells regulated by exogenous gga-miR-3525. These results indicate that gga-miR-3525 may regulate the proliferation and differentiation of SMSCs through the MAPK signaling pathway. Of course, the details of how gga-miR-3525 regulates the p38-MAPK pathway by inhibiting PDLIM3 require more in-depth research, including the expression changes of related proteins in the p38-MAPK pathway. The experiment of this research is not perfect, but this is also the direction of our efforts in the future research process.

## 4. Materials and Methods

### 4.1. Ethics Standards

All animal experimental procedures in this study were approved by the Animal Welfare Committee of Sichuan Agriculture University, and the assurance number is 2019-007 (3 July 2019).

### 4.2. Animals and Samples

Ross 308 broiler were used in this research and obtained from the Sichuan Yuguan Agriculture Co., Ltd. (Suining, Sichuan, China). All collected samples were quickly frozen and ground in liquid nitrogen, and then stored at −80 °C for RNA and protein isolation.

### 4.3. Cell Culture

SMSCs were initially isolated and cultured from the breast muscles of 4-day-old ROSS-308 chickens. After removing the skin and bones, the breast muscles were cut with scissors and digested with 0.1% type I collagenase (Sigma Chemical Co., St. Louis, MO, USA) and 0.25% trypsin (Gibco, Grand Island, NY, USA) to release the cells. The cell suspension was then filtered and subjected to Percoll density centrifugation to isolate satellite cells. Add 10% growth medium (GM: Dulbecco’s modified Eagle medium (DMEM) (Gibco), +10% fetal bovine serum (Gibco), +0.2% penicillin/streptomycin (Invitrogen, Carlsbad, CA, USA)) to the isolated satellite cells to cultivate. When satellite cells confluent in growth medium to 70–80% confluence, the differentiation medium (DM: DMEM +2% horse serum (Hyclone, Logan, UT, USA) was used to induce differentiation. Chicken embryo fibroblast cell line DF-1 was used for dual luciferase reporter gene determination and cultured in 10% GM. The cells were cultured in a constant temperature and humidity cell incubator (Thermo Scientific, San Jose, CA, USA) (5% CO_2_ humid atmosphere, 37 °C), and the medium was refreshed every 24 h.

### 4.4. Construction and Transfection of Plasmid and RNA Oligonucleotides

MiRNA related reagents include gga-miR-3525 inhibitors, negative inhibitors, gga-miR-3525 mimics and negative mimics were purchased from RiboBio (RiboBio, Guangzhou, China). Three siRNAs for chicken PDLIM3 were designed and purchased from GenePharma (GenePharma, Shanghai, China). Two restriction enzymes, BamHI and XhoI, were used to construct the vector pcDNA3.1-PDLIM3 (Sangon Biotech, Shanghai, China). The detailed sequence is shown in Table 1.

The gga-miR-3525 mimics, inhibitors, si-PDLIM3 or pcDNA3.1-PDLIM3 were diluted using Lipofectamine^®^ 3000 (Invitrogen) and Opti-MEM^®^ (Gibco) medium according to the manufacturer’s instructions, then transfected into satellite cells. When cells are cultured in GM for 50–60%, they can be transfected for cell proliferation-related tests. For cell differentiation-related tests, the cell fusion rate needs to reach about 80–90% and then transfected in DM.

### 4.5. Extraction of RNA, Synthesis of cDNA, and Real-Time Quantitative PCR

According to the manufacturer’s instructions, total RNA was extracted from satellite cells using TRIzol reagent (Invitrogen) to detect gene expression. Thermo Scientific™ NanoDrop Lite (Thermo, San Jose, CA, USA) was used to measure the integrity and concentration of RNA. Reverse transcription of mRNA was performed using PrimeScript RT Master Mix Perfect Real Time (Takara, Dalian, China), reverse transcription reactions for miRNA were performed using the One Step miRNA cDNA Synthesis Kit as per manufacturer’s instructions (HaiGene, Haerbin, China).

The real-time PCR primers were designed by Prime Premier 6 (PREMIER Biosoft, CA, USA). The details of the primer sequences are listed in Table 2. The CFX96-TouchTM real-time PCR detection system (Bio-Rad, Hercules, CA, USA) was used to measure the mRNA abundance of each gene. All reactions were repeated three times. The relative expression levels of different qRT-PCR data were analyzed using the 2^−ΔΔCt^ method.

### 4.6. Cell Proliferation

According to the manufacturer’s instructions, Cell Count Kit-8 (CCK-8; Meilunbio, Shanghai, China) was used to measure cell proliferation, and four time points were measured at 12, 24, 48, and 72 h after transfection. The cells were transfected when they reach 40–50%. After 6 h of transfection, it was replaced with fresh GM medium. Then, 24 h after transfection, EDU experiments were performed using C10310 EDU Apollo in vitro imaging kit (RiboBio) according to the instructions. The cells were first incubated with EdU medium for 3 h, and then washed with PBS (phosphate buffered saline). The cells were then fixed with 4% paraformaldehyde and stained with the kit. Three areas were randomly selected under a fluorescence microscope to observe the number of stained cells.

When cells were transfected with siRNA or vector for 48 h, the cells were collected and suspended in 75% ethanol and kept overnight at 20 °C. Then, the cells were incubated with 500 μL of propidium iodide (PI)/RNase staining buffer (BD Biosciences, Franklin Lakes, NJ, USA) at 37 °C for 15 min and detected. Analysis was performed using BD AccuriC6 flow cytometer (BD Biosciences) and Modfit LT 3.2 (Verity Software House, Topsham, ME, USA).

### 4.7. Cell Differentiation

According to the manufacturer’s instructions, the cells were fixed with 4% paraformaldehyde (RiboBio) for immunofluorescence experiments. The detailed experimental method of immunofluorescence has been introduced in depth before [34]. The fixed cells were permeated with 0.5% Triton X-100 at room temperature for 20 min and blocked with goat serum for 30 min. Next, the diluted primary antibody was added and incubated at 4 °C overnight. The diluted secondary antibody was added the next day and incubated at 37 °C for 1 h. Next, stain the nuclei with DAPI (4′,6-diamidino-2-phenylindole) in the dark for 5 min. Three images were taken randomly using a fluorescence microscope (Olympus, Tokyo, Japan). Image-Pro Plus software was used to measure myotube area.

The detailed experimental methods for Western blot was referred to the description of Zhao et al. [35]. The total protein was extracted from the transfected cells using a total protein extraction kit (BestBio, Shanghai, China), and the protein sample concentration was evaluated according to the instructions of the BCA protein quantification kit (BestBio), and the optimal sample size was calculated. Antibodies used in the experiment include: anti-MYHC (Santa Cruz Biotechnology, Dallas, TX, USA; 1:200 dilution), anti-MyoD (Santa Cruz Biotechnology; 1:500 dilution), anti-phospho-p38 MAPK, p38 MAPK (Sigma, USA), and β-Tubulin (ZENBIO, Beijing, China: 1:5000 dilution). β-Tubulin was used as a loading control.

### 4.8. Luciferase Reporter Assay

Fragments of gga-miR-3525, including the binding site of PDLIM3 (150 bp upstream and downstream of the binding site), were amplified and inserted into pEZX-FR02 vectors (GeneCopoeia, USA) at the 3’ end of the Firefly Luciferase gene using restriction enzymes BsiWI and XhoI (TaKaRa, Dalian, China) and T4 DNA ligase (pEZX-PDLIM3-WT). Mutant pEZX-PDLIM3-MT was generated by mutating complementary to the seed region of gga-miR-3525 using mutagenic primers. All constructs were verified by sequence analysis.

### 4.9. Transcriptome Analysis

According to the standard Trizol protocol, after 48 h of transfection with gga-miR-3525 inhibitor and inhibitor NC in SMSCs, Trizol was used to extract total RNA from the cells. The quantity and quality of extracted RNA were analyzed using a Nanodrop NC2000 (Thermo Fisher Scientific) and an Agilent 2100 Bioanalyzer (Agilent Technologies, Santa Clara, CA, USA), respectively. The qualified RNA samples (RIN > 7; 3 independent samples from two groups, respectively) were sent to transcriptome sequencing by Illumina Hiseq 2000 instrument in Beijing Novogene Technology Co., Ltd.

### 4.10. Statistical Analysis

Statistical analysis was performed using SPSS 19.0 Statistics software (SPSS, Inc., Chicago, IL, USA). Each experiment was repeated three times. The data are expressed as the mean standard error of the mean (SEM). Unpaired Student’s *t*-test was used for comparative analysis of the two groups. One-way ANOVA was used for comparative analysis of multiple groups. *p* < 0.05 is considered to indicate statistical significance.

## 5. Conclusions

**Figure 8:**
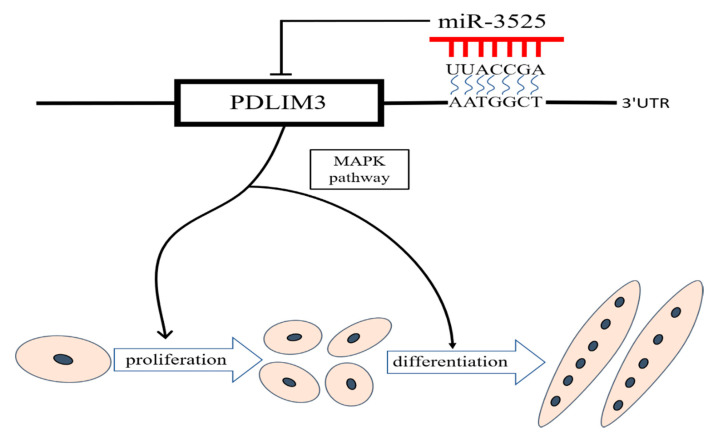
gga-miR-3525-mediated chicken skeletal muscle satellite cell regulatory pathway model.

In this study, we confirmed that gga-miR-3525 inhibits the development of SMSCs by targeting PDLIM3 and regulating the p38/MAPK signaling pathway, thus inhibiting the proliferation and differentiation of SMSCs (Figure 8). We elaborated on the mechanism by which gga-miR-3525 exerts its regulatory effect by suppressing target genes. This model may help us better understand the role of gga-miR-3525 and other miRNA in myogenesis.

## Figures and Tables

**Table 1:** RNA oligonucleotide and plasmid construction in this article.

Name	Sequence (5′-3′)
miR-3525 mimic	CAGCCAUUCUGCGAUUCUGUGA ACAGAAUCGCAGAAUGGCUGUU
Negative mimic	UUGUACUACACAAAAGUACUG
miR-3525 inhibitor	UCACAGAAUCGCAGAAUGGCUG
Negative inhibitor	CAGUACUUUUGUGUAGUACAA
si-PDLIM3-333	GCAAAGCAAAUCCCUACAATT UUGUAGGGAUUUGCUUUGCTT
si-PDLIM3-457	GGUAGUGAGCUCCUCCUAUTT AUAGGAGGAGCUCACUACCTT
si-PDLIM3-791	GGGAGUGGAAUUCUAGGAATT UUCCUAGAAUUCCACUCCCTT
si-NC	UUCUCCGAACGUGUCACGUTT ACGUGACACGUUCGGAGAATT
pcDNA3.1-PDLIM3	GACACAAGCTTGCCACCATGCCACA GTGTCCTCGAGCTAGCATTTTGGGTA

**Table 2:** Primers used for quantitative real-time PCR.

Gene	Primer Sequences (5′-3′)	Product Size (bp)	TM (°C)
β-actin	F: GTCCACCGCAAATGCTTCTAA R: TGCGCATTTATGGGTTTTGTT	78	58
MyoG	F: CGTGTGCCACAGCCAATG R: CCGCCGGAGAGAGACCTT	63	60
MyoD1	F: GCCGCCGATGACTTCTATGA R: CAGGTCCTCGAAGAAGTGCAT	66	60
MYHC	F: GAAGGAGACCTCAACGAGATGG R: ATTCAGGTGTCCCAAGTCATCC	138	60
PDLIM3	F: AAGCACCTGTAACAAAGATA R: GCCCTCCACAAAGAAGTAGC	188	55
MYBPH	F: ATCCGCCTACCTCGTCAG R: GGCTGGTTGTCCTTGGTC	122	58
MYL10	F: TGTTGCTTAACCTCTTGCTTT R: TACCAAATGCTCTTCCCAGT	89	56
MYL3	F: GAAGAACCCAAACCAGCA R: CCCAAAGCCCTCAAGAC	200	55
TNNT3	F: GGCTGAGAAGGAGAAGGAG R: GCTGTATGAGGCACCCA	136	56
MYMK	F: TCCCCACCATCAGCATC R: GCATGAAACATAGCACCGA	129	56
MAPK13	F: TTCCTGAGTCGTGTTTGGT R: GGGGCATGGCTGTAGTAA	145	56
CAV3	F: GTGCCCTGCATCAAGAG R: CGCAGCATAACCCTGAC	134	55
CRK	F: CACTCCGCTCCCTAACC R: CCTTCCCACTGACCACTC	150	56
MAP2K3	F: GCCTATGGTGTGGTGGAGA R: AAGCAGTCAACCGTCCTCA	143	58
MAP2K5	F: GGCCAGATGAATGAACAAG R: GCCAGGATTTTCCCACTA	104	54
miR-3525	F: CAGCCATTCTGCGATTCTGTGA R: CAGGTCCAGTTTTTTTTTTTTTT	–	56
U6	F: GGGCCATGCTAATCTTCTCTGTA R: CAGGTCCAGTTTTTTTTTTTTTT	–	56
